# Tools enabling the elucidation of molecular pathways active in human disease: Application to Hepatitis C virus infection

**DOI:** 10.1186/1471-2105-6-154

**Published:** 2005-06-20

**Authors:** David J Reiss, Iliana Avila-Campillo, Vesteinn Thorsson, Benno Schwikowski, Timothy Galitski

**Affiliations:** 1 Institute for Systems Biology, 1441 N. 34^th ^Street, Seattle, WA 98103, USA; 2 Institut Pasteur, 25–28 Rue du Dr. Roux, 75724 Paris CEDEX 15, France

## Abstract

**Background:**

The extraction of biological knowledge from genome-scale data sets requires its analysis in the context of additional biological information. The importance of integrating experimental data sets with molecular interaction networks has been recognized and applied to the study of model organisms, but its systematic application to the study of human disease has lagged behind due to the lack of tools for performing such integration.

**Results:**

We have developed techniques and software tools for simplifying and streamlining the process of integration of diverse experimental data types in molecular networks, as well as for the analysis of these networks. We applied these techniques to extract, from genomic expression data from Hepatitis C virus-infected liver tissue, potentially useful hypotheses related to the onset of this disease. Our integration of the expression data with large-scale molecular interaction networks and subsequent analyses identified molecular pathways that appear to be induced or repressed in the response to Hepatitis C viral infection.

**Conclusion:**

The methods and tools we have implemented allow for the efficient dynamic integration and analysis of diverse data in a major human disease system. This integrated data set in turn enabled simple analyses to yield hypotheses related to the response to Hepatitis C viral infection.

## Background

DNA microarrays have been applied with much success to study genomic patterns of gene expression across many organisms. It has become widely acknowledged that to extract hypotheses from these data, there are advantages to the integration of orthogonal sources of information, notably, molecular-interaction data [1]. Hypotheses derived from genomic-expression data typically involve pathways of metabolic and molecular information flow, and complex cellular processes and structures, formed by multiple interacting molecules. However, commonly these molecular interactions are gleaned *ad hoc *from the literature.

In model organisms such as *Saccharomyces cerevisiae*, integrative systems-biology approaches to genomic-expression analysis have developed and employed sophisticated methods for the computational extraction of biological knowledge. Examples include: biological module identification and abstraction [2]; discovery of regulatory networks [3,4]; and identification of active pathways in networks [5]. A hallmark of these advanced methods is the integration of diverse genome-scale data sets, in particular, the combination of genomic-expression data and molecular-interaction data. Another common characteristic of these methods is the use of graphs (vertices and edges, or nodes and links) to represent such integrated data. Graphical methods are highly intuitive. Also, the formalism of the graph facilitates the development and application of graph algorithms and machine-learning techniques to extract information.

In studies of human disease, a limited repertoire of computational techniques, including ANOVA, hierarchical clustering, and discriminant analysis, has been applied to extract information from genomic-expression data derived from human tissues. Until recently, a critical barrier has been a lack of large-scale machine-readable sources of high-quality human molecular interaction data. Using a combination of artificial-intelligence methods and expert human curation, several efforts have made substantial progress in amassing, from the literature, databases with large numbers (greater than 14000) of human molecular interactions. These include the Human Protein Reference Database (HPRD) [6,7], the Biomolecular Interaction Network Database (BIND) [8,9], the Database of Interacting Proteins (DIP) [10,11], and the Transcription Factor Database (Transfac) [12]. Thus, the bottleneck has now shifted to the efficient integration of these data to enable the application of advanced network-based analysis and modelling methods. For this work, we have implemented solutions to this bottleneck and applied them to a set of genomic-expression data derived from biopsies of human liver tissue infected with Hepatitis C Virus (HCV) [13]. About 3% of all humans are infected with HCV [14], and currently no vaccine exists. Chronic viral hepatitis C results in liver fibrosis and cirrhosis in about 20% of those infected [15]. Liver transplant is often required.

Specifically, we have developed two software tools, *InteractionFetcher *and *CytoTalk*, that function as plug-ins for *Cytoscape*, an open-source, platform-independent environment for the visualization and analysis of biological networks [16,17]. *InteractionFetcher *and *CytoTalk *simplify the integration and analysis of interaction data (and other data types) with genomic-expression data. To demonstrate their utility, we applied them to generate and analyze a large network of human molecular-interaction pathways that are putatively active during the infection of human liver tissue with HCV.

## Implementation

### *InteractionFetcher*, a *Cytoscape *plug-in

*InteractionFetcher *dynamically retrieves remote biological information for selected nodes in the current network within *Cytoscape*. The plug-in requests biological data via the XML-RPC protocol [18] from a remote server, which retrieves the requested information from an SQL database and passes it back to the plug-in. The plug-in then adds the retrieved information to the current network as additional nodes, edges, and/or attributes. Currently implemented data types include: protein/gene synonyms, orthologs, sequences (gene/protein/upstream), and interactions/associations. Some of this information can be obtained via integrated queries. For example, retrieved gene/protein synonym information may be used to increase the number of molecular interactions that are found. Currently-available interaction-data sets include HPRD [6,7], BIND [8,9], DIP [10,11], and several other predicted interaction and co-expression data sets [19-21]. Many options are available, including the ability to do cross-species queries, using ortholog information from Homologene [22] among species including *H. sapiens*, *M. musculus*, *S. cerevisiae*, *C. elegans*, and *D. melanogaster*. For example, if two proteins in *H. sapiens *have not been observed to interact, but both of their orthologs in *S. cerevisiae *are known to interact, then an *inferred interaction *(also known as an interolog) can be added to the network. Moreover, the tool allows for easy viewing of the source database's web page or linked PubMed abstract(s) describing each fetched interaction. Because the source code for both the client and server of this plug-in are available, we hope that the capabilities of plug-ins such as these can be expanded by other researchers to include, for example, experimental data (such as mRNA expression levels), metabolic information, or functional annotations. *Cytoscape*, the *InteractionFetcher *and related plug-ins, plus all server-side software are open-source and may be obtained at our laboratory web site [23] or at the *Cytoscape *web site [17].

### *CytoTalk*, a *Cytoscape *plug-in

*CytoTalk *enables a *Cytoscape *user to dynamically interact with and manipulate the current network in a *Cytoscape *window from an external process. This plug-in runs an internal XML-RPC [18] server that enables the currently-displayed network and its various attributes to be manipulated from an external client that is XML-RPC-capable. Example clients may include Perl and Python scripts, scripts written in the *R *statistical language [24], UNIX shell scripts, C or C++ programs, or Java processes. It moreover expands the developmental possibilities of *Cytoscape *plug-in developers by allowing other plug-ins to be written in these languages. The external process may be run on the same machine as *Cytoscape*, or anywhere else on an accessible network. The open-source *CytoTalk *and *Cytoscape *software as well as example *CytoTalk *clients in *Perl*, *Python*, and *R *may be obtained at our laboratory web site [23] or at the *Cytoscape *web site [17].

## Results and discussion

### Gene-expression data

For our study, we utilized expression data derived from 28 liver biopsies collected by [13] from 11 HCV-positive liver transplant patients, between 1 and 24 months post-transplant. Since roughly 50% of HCV+ liver transplant patients become re-infected during the two years after receiving their new livers [25], these biopsies provide a unique model for tracking the changes in gene expression during HCV infection [13]. To compare gene-expression patterns in liver tissue before and after infection with HCV, [13] collected 28 post-transplant liver biopsies, plus pre-transplant control biopsies, from 11 HCV+ liver-transplant patients. Liver biopsies were obtained at intervals of 3 to 6 months, between 1 and 24 months post-transplant [13]. These samples contain a mixture of cell types including hepatocytes, hepatic stellate cells, Kupffer cells (liver macrophages), in addition to various blood cells [13]. mRNA expression ratios of about 7000 genes were measured relative to a common reference pool of pre-transplant biopsies. Using Rosetta *Resolver*(R) software [26], the data were normalized and transformed to log_10 _ratios, and p-values were computed for expression difference from the reference pool. The measurements showed a high degree of patient-to-patient variation. Most of the genes (5968) were significantly expressed (*p *<10^-7^) in at least one of the 28 samples. The research of [13] involved genomic-expression data derived from human subjects.

### Construction of the molecular-interaction scaffold

**Figure 1:**
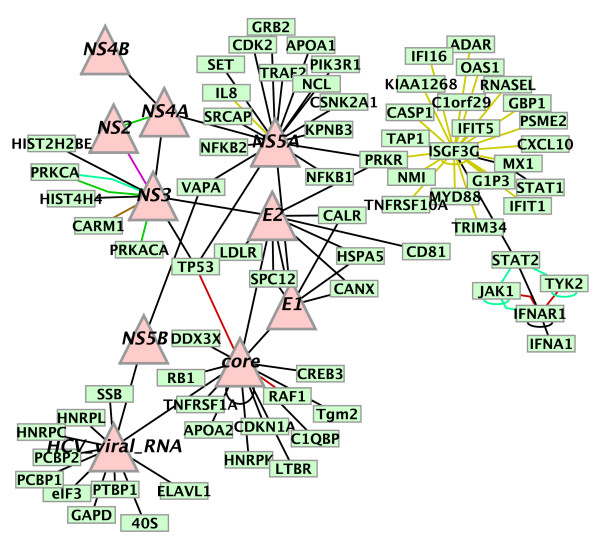
**Network of interactions among HCV-encoded molecules and host proteins.**Triangular nodes represent HCV-encoded molecules. Host molecules are square nodes. Edges represent molecular interactions of several types: black for protein-protein, yellow for protein-DNA, light-green for phosphorylations, red for activations, dark-green for repressions, purple for covalent interactions, brown for methylations. Sources: [14, 15 and references therein].

We sought to generate a network of molecular pathways that are active (either induced or repressed) in HCV-infected human liver cells. The effects of HCV infection are likely to be complex, and the presence of contaminating blood cells and mixtures of various cell types in the biopsy samples will add further complexity. In order to emphasize the network interface between viral molecules and human molecules, we initiated network construction with a small "seed" network of interactions among HCV-encoded molecules and between HCV-encoded and host-encoded proteins. Interaction data were curated from review articles ([27,28], and references therein). The seed network also included the JAK-STAT interferon-response pathway that is known to play a role in the response to HCV infection [29]. This set comprised 106 interactions between 86 macromolecules (proteins and the viral RNA). The proteins were, when possible, cross-referenced to RefSeq protein identifiers [30,31]. Figure 1 shows the seed network visualized using *Cytoscape *[16,17]. This network is available for exploration and analysis via *Cytoscape *at our laboratory web site [23].

**Figure 2:**
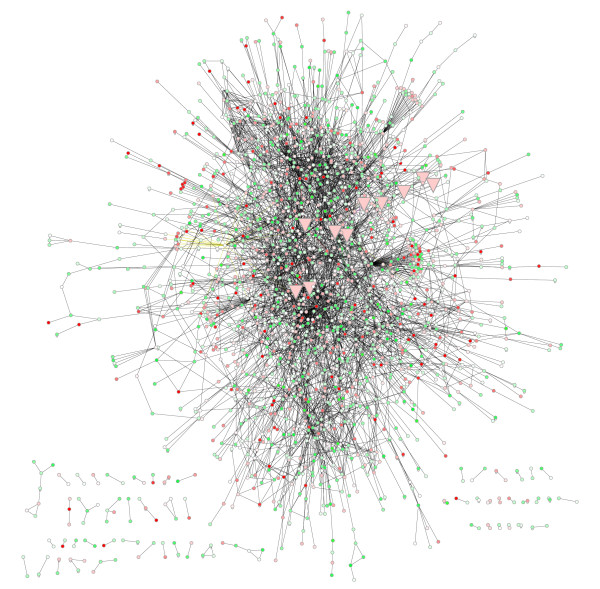
**Network of interactions among proteins implicated by genomic expression data. **Genes were implicated by expression profiling of HCV-infected liver biopsy data [13]. The network of interactions was assembled from external databases HPRD [6, 7] and BIND [8, 9], and automatically integrated using *InteractionFetcher*.

The seed network was expanded to a full "scaffold" network using the 5968 genes implicated by the genomic-expression data and large-scale molecular-interaction data sets in public databases by searching for interactions among the 5968 expressed genes and the molecules in the seed network. To automate the construction of the scaffold network, we implemented a *Cytoscape *plug-in, *InteractionFetcher*, for dynamic retrieval of molecular interactions and binding partners via the Internet. *InteractionFetcher *rapidly adds interactions among molecules of interest in a network. In addition, it may be used to iteratively expand a network through "in silico pull-down" of molecules that are currently not present in the network but are known to interact with molecules that are present. Using this plug-in, we were able to integrate as many as 15,000 interactions among the proteins implicated by the HCV expression-data set and seed-network proteins (among the available interaction data sets, which include HPRD [6,7], BIND [8,9], DIP [10,11], PreBIND [32,33], and several other predicted and co-expression data sets; see Methods). However, for this paper, we restricted our search to individually curated human-only protein interactions from HPRD and BIND, resulting in a scaffold network of 4,592 unique interactions among 1,950 molecules (Figure 2). This network is available for exploration and analysis with *Cytoscape *at our laboratory web site [23], which also allows for easy viewing of additional information provided by *InteractionFetcher*, such as each interaction's source database web page and PubMed abstract identifier(s).

### Computational analysis of integrated gene-expression data and molecular-interaction data

A useful method of integrated analysis of expression data within an interaction network is the *ActivePaths *algorithm [5]. This method identifies contiguous pathways or subnetworks that are active (induced or repressed relative to randomly selected subnetworks) in subsets of the expression data. We applied this algorithm, which is available as a *Cytoscape *plug-in, to the scaffold network. Due to the high order (number of vertices) and size (number of edges) of the scaffold network, it was necessary to iteratively apply the algorithm, as suggested by the developers, to obtain increasingly smaller active subnetworks until they contained fewer than 100 nodes. The resulting four active subnetworks contained between 40 and 121 interactions. Because there were overlaps among these four highest-scoring active subnetworks, we combined them into a single fully connected active subnetwork.

Additional analyses were performed by selecting scaffold subnetworks that are significantly active and/or co-regulated in temporal subsets of the microarray data. Because the scaffold network is not differentiated with regard to tissues, cell types, or cellular state, and the biopsy samples from which the expression data were derived likewise contain mixtures of cell types and other contaminants, the information in the active scaffold network does not, by itself, answer the questions we are addressing. To increase our chance of identifying pathways that might be modulated in response to HCV infection, we performed a differential analysis of the scaffold network, to identify subnetworks that become active more than eight months after transplant. This choice of cut-off was made to nearly-evenly divide the expression data into two halves (those from biopsies prior to, and after, eight months post-transplant), and by performing a differential analysis we can hope to subtract out some of the effects of the transplant and post-transplant immune response signals from those of HCV reinfection and progression.

We used the *R *statistical environment [24] to perform this analysis. Because *R *is external to *Cytoscape*, we developed a plug-in, called *CytoTalk*, that enables a user of *R *(or a wide variety of other environments or languages; see Methods) to interactively query and modify *Cytoscape *networks, thereby greatly expanding the analytical capabilities available to users of *Cytoscape*. We used *R *with *CytoTalk *to select the proteins and interactions implicated by specific statistical queries on the expression data. This enabled us to extract a subnetwork of genes that were significantly induced or repressed with |log_10_(ratio)| > 0.4 in biopsies obtained more than 8 months after transplant. The proteins encoded by these genes form a "late-active" network. We similarly extracted an "early-active" network encoded by genes that were active in biopsies obtained earlier than 8 months after transplant. We compared these two networks and identified an "only-late-active" subnetwork that was not active prior to eight months, but was active afterward. The expectation is that this "only-late-active" subnetwork will contain pathways from the "late-active" network that are activated in response to Hepatitis C virus re-infection, while pathways from the "early-active" network that may contain pathways activated as a result of the transplant are removed.

**Figure 3:**
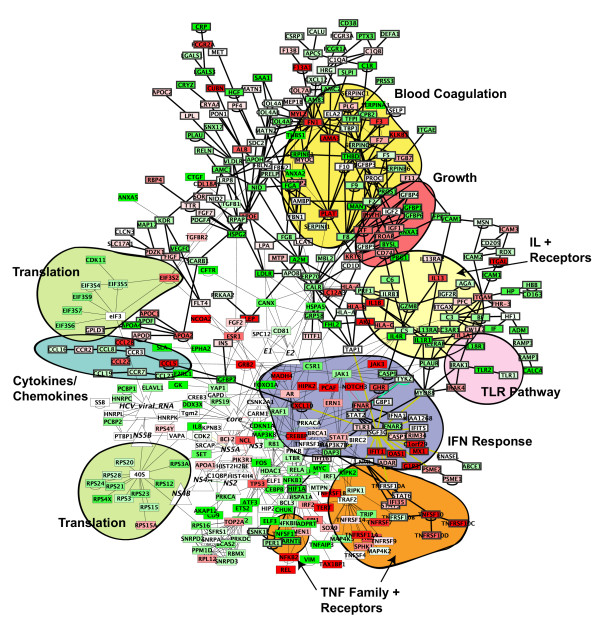
**Composite network of molecular pathways active in HCV-infected liver tissue. **The network in Figure 1 was combined with active subnetworks from the network in Figure 2. The active subnetworks were identified by active-paths analysis ([5]; bold nodes and edges) and by identifying the subnetworks that changed most significantly in expression with time after transplant. Nodes (genes) colored red were induced in the expression data of biopsies from 8 months or more post-transplant; green nodes were repressed. Areas that contain differentially active pathways or subnetworks, as described in the text, are highlighted.

In Figure 3, we have integrated the seed network, the composite active-paths network, and the "only-late-active" network into one network. This network is available for exploration and analysis in *Cytoscape *at our laboratory web site [23]. Genes that were induced on average after 8 months following transplant are indicated with a red colour. Genes that were repressed are green. We have highlighted the nodes and edges of the composite active-paths subnetwork in "bold". The network in Figure 3 is significantly over-represented with genes of several biological processes, as annotated by the Gene Ontology Consortium Database [34,35]; using the *BioDataServer *tool in *Cytoscape*, and computed in *R *via *CytoTalk*, using the Bonferroni-corrected hypergeometric distribution. Among these include blood coagulation (*p *= 10^-11^), immune response (*p *= 10^-7^), proteolysis and peptidolysis (*p *= 10^-5^), lipid transport (*p *= 10^-3^), and complement activation (*p *= 10^-2^). In addition, nearly the entire JAK-STAT interferon-response signalling pathway is activated in this network.

The visualization in Figure 3 enables one to identify these pathways and see whether they are "turning on" (red) or "turning off" (green) in the expression data. For example, the blood coagulation pathway is active in the expression data, although (as is to be expected with large and complex pathways) not coherently induced or repressed. The interferon-response pathway and genes activated by ISGF are clearly induced, probably due in part to the immune response to viral infection and partly in response to standard treatment of HCV-positive patients with interferon-alpha. Also, genes encoding the Toll-like receptors TLR1 and 2, as well as the downstream signalling pathway connecting them, through MYD88, to the interferon-response pathway appear to be repressed. TLRs 1 and 2 are known viral detection receptors; it is known that TLR2 detects HCV [36]. The interleukin receptor IL1R1, upstream of MYD88, is also repressed along with other IL receptors, whereas IL1A and B are induced. Additionally, we see that many apoptosis-related genes encoding TNF, TNF receptors, and TNF-signalling factors, are activated, whereas growth factors (IGF and connected pathways), and cell cycle and translation-related pathways (*e.g*. CDKN and connected pathways) are repressed. Ignoring the observed responses that are likely due to by-products of the biopsy process (*e.g*. the blood coagulation pathway), the active pathways observed are jointly consistent with a large-scale response of complex molecular pathways to viral infection: hepatic cell reproduction is repressed and programmed cell death is induced.

Finally we note that a visual inspection of the network suggests that many of the proteins that bind directly to HCV-encoded molecules (*i.e*., are their first neighbours in the network) appear on average to be down-regulated relative to the rest of the network. Statistical analysis of the data supports this suggestion. As computed via *CytoTalk *and *R*, about 80% of the first neighbours of the viral RNA and proteins are down-regulated in the network of Figure 3, compared to 35% of the remaining genes in the network (*p *= 0.0029). This finding suggests two non-exclusive possibilities: genes encoding HCV-neighbour proteins are targets of host regulatory mechanisms counteracting viral replication; or they are targets of virus-encoded regulatory mechanisms that sabotage anti-viral defences.

## Conclusion

The methods and software tools described here enable the efficient dynamic integrated analysis of diverse data in a major human-disease system. The results show the utility of integrating large-scale human molecular-interaction databases with genomic expression data. This approach is useful for the extraction of biological hypotheses, because it allows us to focus on groups of genes that are not only apparently active in the expression data, but are also functionally associated based on other data, such as molecular interactions. Thus, information that is not restricted to any one data type can be obtained. Moreover, our analyses suggest how various pathways act in concert, and serves as a large-scale window into the genomic response to HCV infection of liver cells. Because the tools and methods we have described are data-type-neutral, there is the prospect of further data integration for a more complete systems-biological approach to understanding viral infection and response mechanisms. The integration of additional, orthogonal sources of information such as detailed clinical data will enable quantitative associations of clinical variables with the activities of molecular pathways and processes.

## Availability and requirements

• Project name: *InteractionFetcher*, *SynonymFetcher*, *HomologFetcher*, and *CytoTalk*: plug-ins for *Cytoscape*

• Project home page: 

• Operating system(s): Platform independent

• Programming language: Java

• Other requirements: Java 1.4 or higher

• License: GNU LGPL

• Any restrictions to use by non-academics: license required for access to HPRD interactions (see [7])

## Authors' contributions

DJR: Development of *InteractionFetcher*, *CytoTalk *and associated server-side software and databases, construction of seed and scaffold network, analyses of active pathways, functional analyses, manuscript preparation. IA: Expression data processing, *ActivePaths *and functional analysis. VT: Construction of seed and scaffold networks, functional analysis and biological interpretation. BS: Project conception and planning. TG: Guidance, construction of seed network, manuscript preparation.
